# Dynamics of Etiolation Monitored by Seedling Morphology, Carotenoid Composition, Antioxidant Level, and Photoactivity of Protochlorophyllide in *Arabidopsis thaliana*

**DOI:** 10.3389/fpls.2021.772727

**Published:** 2022-02-22

**Authors:** Pawel Jedynak, Kamil Filip Trzebuniak, Magdalena Chowaniec, Piotr Zgłobicki, Agnieszka Katarzyna Banaś, Beata Mysliwa-Kurdziel

**Affiliations:** ^1^Department of Plant Physiology and Biochemistry, Faculty of Biochemistry, Biophysics and Biotechnology, Jagiellonian University, Kraków, Poland; ^2^Department of Plant Biotechnology, Faculty of Biochemistry, Biophysics and Biotechnology, Jagiellonian University, Kraków, Poland

**Keywords:** etiolation, *Arabidopsis thaliana*, *lut2* mutant, protochlorophyllide, Pchlide_654_/Pchlide_633_, light-dependent protochlorophyllide oxidoreductase, *LPOR*

## Abstract

Although etiolated *Arabidopsis thaliana* seedlings are widely used as a model to study the de-etiolation process, the etiolation itself at the molecular level still needs elucidation. Here, we monitored the etiolation dynamics for wild type *A. thaliana* seedlings and lutein-deficient (*lut2*) mutant between 2 and 12 days of their growth in the absence of light. We analyzed the shape of the apex, the growth rate, the carotenoids and protochlorophyllide (Pchlide) accumulation, and the light-dependent protochlorophyllide oxidoreductase (*LPOR*) transcripts. Differences concerning the apical hook curvature and cotyledon opening among seedlings of the same age were observed, mostly after day 6 of the culture. We categorized the observed apex shapes and presented quantitatively how distribution among the categories changed during 12 days of seedling growth. The Pchlide_654_/Pchlide_633_ ratio, corresponding to the amount of the photoactive Pchlide, was the highest in the youngest seedlings, and decreased with their age. *LPORA*, *LPORB*, and *LPORC* transcripts were detected in etiolated seedlings, and their content decreased during seedling growth. Expression of *SAG12* or *SAG13* senescence markers, depletion in antioxidants, and excess ion leakage were not observed during the etiolation. Lack of lutein in the *lut2* mutant resulted in slow Pchlide accumulation and affected other xanthophyll composition.

## Introduction

Light is a driving force for photosynthesis, enabling the autotrophic lifestyle of plants. Angiosperms, the most developed photosynthetic organisms, use light not only to power photosynthesis but also to manage plant growth. In the absence of light, angiosperm seedlings undergo etiolation that involves morphological, physiological, and biochemical processes that are evolutionary adaptations for efficient resource management, increasing the chance of survival (Armarego-Marriott et al., 2020). These processes, in the case of dicotyledon plants, include rapid hypocotyl elongation and formation of the apical hook that wraps the closed cotyledons down (Wei et al., 1994; Nemhauser, 2008). This is, for example, the case of seeds buried deeply in soil that are devoid of light and can rely only on resources stored in dicot cotyledons (or monocot endosperm) as a source of energy for their development.

In etiolated seedlings, at the cellular level, proplastids develop into etioplasts instead of chloroplasts (typical for light-grown plants). A paracrystalline lipid structure, called prolamellar body (PLB), is characteristic for etioplasts (Solymosi and Aronsson, 2013; Armarego-Marriott et al., 2020; Solymosi and Mysliwa-Kurdziel, 2021). The biosynthesis of some protein subunits of photosynthetic complexes is inhibited, and the biosynthesis of chlorophyll (Chl) pauses at the stage of protochlorophyllide (Pchlide) formation, and they both require light to continue (Tanaka et al., 2011; Pipitone et al., 2021). In angiosperms, the conversion of Pchlide to chlorophyllide (Chlide), which is the penultimate reaction of the Chl biosynthesis pathway, is catalyzed only by the light-dependent protochlorophyllide: NADPH oxidoreductase (LPOR; EC 1.3.1.33) that requires light for its activity (Gabruk and Mysliwa-Kurdziel, 2015; Heyes et al., 2021; Solymosi and Mysliwa-Kurdziel, 2021). LPOR is the major protein found in isolated PLBs (Lindsten et al., 1988; von Zychlinski et al., 2005; Blomqvist et al., 2008), and vast majority of LPOR is localized in PLBs (Ryberg and Sundqvist, 1982; Ikeuchi and Murakami, 1983). Pchlide and LPOR accumulate in etioplasts as ternary Pchlide: LPOR: NADPH complexes along with unbound Pchlide. Those Pchlide forms can be distinguished with absorption and fluorescence spectroscopy at 77 K that have been thoroughly summarized in many reviews (e.g., Schoefs and Franck, 2003; Belyaeva and Litvin, 2007, 2014; Solymosi and Mysliwa-Kurdziel, 2021). In particular, fluorescence bands of Pchlide: LPOR: NADPH complexes and that of unbound Pchlide have maxima at about 654 and 633 nm, respectively. Pchlide bound to the ternary complexes is reduced to Chlide immediately upon illumination and thus is called photoactive Pchlide. The unbound Pchlide, described as non-photoactive, stays unreduced after a single-flash illumination, and serves as the substrate for Chl biosynthesis on a longer time scale.

Three isoforms of LPOR were identified in *Arabidopsis thaliana* (Armstrong et al., 1995; Su et al., 2001). LPORA predominantly accumulates in dark-grown seedlings and at the beginning of de-etiolation that is a light-induced transition of etiolated seedlings to green ones. LPORB is present throughout the whole plant life, whereas LPORC accumulates in the presence of light (Runge et al., 1996; Frick et al., 2003; Paddock et al., 2012). The expression of *LPORs* in *A. thaliana* is co-orchestrated by multiple molecular regulators with a crucial role of the COP/DET/FUS (Constitutive Photomorphogenic/De-Etiolated/Fusca) proteins (Gabruk and Mysliwa-Kurdziel, 2015). These factors were indicated as major negative regulators of photomorphogenesis, therefore promoting etiolation in the absence of light (McNellis et al., 1994; Kwok et al., 1996). Light activates photoreceptors, phytochromes and cryptochromes, leading to rapid deactivation of COP/DET/FUS. The detailed regulation of the etiolation is still under debate, and little is known about the role of carotenoids in this process. In dark-grown seedlings, the accumulation of carotenoids is partially inhibited (Toledo-Ortiz et al., 2014), although antioxidative carotenoids are beneficial during the de-etiolation process (Gillham and Dodge, 1985; Rodríguez-Villalón et al., 2009a). Carotenoids are important for the assembly of the PLB structure in etioplasts (Park et al., 2002; Cazzonelli et al., 2020), especially β,β-xanthophylls (Bykowski et al., 2020).

Lutein, an α,β-xanthophyll, is the predominant carotenoid in etiolated *A. thaliana* (Park et al., 2002; Cazzonelli et al., 2009; Mysliwa-Kurdziel et al., 2012; Bykowski et al., 2020) but its physiological role in dark-grown plants is poorly understood. It was shown that lutein content in etiolated seedlings varied among different *A*. *thaliana* ecotypes, and corresponded to the Pchlide content (Mysliwa-Kurdziel et al., 2012), suggesting co-regulation of accumulation of both pigments. Using lutein-deficient *A. thaliana* mutant (*lut2*) as a model, we reveal how the lack of lutein (and thus a reduced overall carotenoid content) affects the accumulation of other pigments, in particular Pchlide, during the etiolation of seedlings. We also investigated the relative content of Pchlide: LPOR: NADPH complexes and the accumulation of *LPOR* transcripts as key components providing an efficient de-etiolation. In light-grown plants, the lack of lutein is compensated by increase of β,β-xanthophylls content (Pogson et al., 1996), but we found no information if such phenomenon occurs in darkness.

Investigation of the dynamics of etiolation process was the other purpose of this study, because dark-grown seedlings of *A. thaliana* are a widely used model to study the de-etiolation process, especially mechanisms of chloroplast development, cell signaling, and plant growth regulation (Jedynak et al., 2014; Armarego-Marriott et al., 2020). However, the etiolation itself has not been yet fully elucidated at the molecular level. What is more, etiolation is treated as a static state rather than a dynamic physiological process. During our work with dark-grown *A. thaliana*, we noticed that the population of etiolated seedlings is heterogeneous concerning the seedling appearance, and that this heterogeneity undergoes changes with time. In this work, we examined large population of etiolated *A*. *thaliana* seedlings and monitored the etiolation process from the very beginning (2 days) up to 12 days of seedling growth in darkness. We analyzed seedling morphology concerning the shape of the apex, biochemistry related to Pchlide and carotenoid accumulation, and verified whether senescence or stress is induced by the lack of light.

## Materials and Methods

### Plant Material

Seeds of *A. thaliana* Col-0 (wild type; WT) and *lut2* mutants [SALK_019364C (Alonso et al., 2003), purchased from the Nottingham Arabidopsis Stock Centre – NASC] were used for experiments. The seeds used in the experiments were obtained from plants grown in controlled conditions (22 ± 2°C), 80% humidity, 12 L: 12D photoperiod at light intensity of 70 to 100 μmol photons m^–2^s^–1^ (Sanyo; Sylvania, Luxline plus). Parent *lut2* plants used in this experiment were homozygous, confirmed by genotyping (Supplementary Data 1).

### Growth Conditions

A targeted portion of seeds, about 6 mg per one culture plate, was weighed on an analytical balance and surface-sterilized with 3% hypochlorite and 0.1% Triton X-100 (Serva, Germany) for 7 min. Then, they were washed 6–10 times with sterile, deionized water. The seeds were sown on a plant culture plate (100 × 40 mm, SPL Lifesciences Co. LTD., Korea) with a Murashige and Skooge (pH 5.7) medium (Duchefa Biochemie, Harlaam, Netherlands), containing 1% agar (without sucrose supplementation) and irradiated with ambient white light for 2 h to improve and synchronize germination. Subsequently, plates were wrapped in a double layer of aluminum foil and placed at 4°C for 24 h (stratification). After that, seedlings were grown at 22 ± 2°C in darkness (a lockable room-size-growing chamber with no windows, no light sources, a double door, air conditioning, and precise temperature control) for 2, 3, 4, 6, 8, and 12 days. Then, the seedlings were collected, gently dried with a paper towel to remove any water droplets, and weighed.

Harvesting of the seedlings and all preparations of samples for analyses described below were performed under scattered dim green safe light, which was previously tested and did not induce chlorophyll biosynthesis in the seedlings (see Supplementary Data 4).

### Carotenoid and Pchlide Determination

Carotenoid and Pchlide were extracted from weighted seedlings (about 100 mg) by homogenizing in small aliquots of 100% acetone with addition of CaCO_3_ (Chempur, Piekary Slaskie, Poland) for buffering. The final volume of the extract was 2 ml. The extract was centrifuged (13,000 *g*, 3 min Eppendorf Mini Spin^®^ centrifuge), and the supernatant was taken for pigment analysis. Total carotenoid content was determined spectrophotometrically (UV-VIS spectrophotometer Jasco V-650, JascoCo, Japan) according to Lichtenthaler (1987) and normalized to seedling fresh weight (FW) or mass of sown seeds. For estimation of the total Pchlide content, the florescence spectra (Perkin Elmer LS-55B, United Kingdom) of the supernatant was measured in quartz cuvettes (Hellma^®^, Sigma-Aldrich; St. Louis, MO, United States) at room temperature. The excitation wavelength was 440 nm, and the emission in the range between 600 and 700 nm was recorded (slits – 10 nm; scan speed, 100 nm/min, without additional filters) with background subtraction. The relative Pchlide content was taken as the intensity of Pchlide fluorescence peak (at 633 nm) and normalized to seedling FW or mass of sown seeds.

### Measurement of the Pchlide_654_/Pchlide_633_ Ratio

The cotyledons from about 50 mg of etiolated seedlings were cut off and briefly homogenized with a micropestle in a 1.5-ml centrifuge vial using 150 μl of an ice-cold 25-mM HEPES-NaOH buffer (pH 7.5), supplemented with D-sorbitol (0.4 M), EDTA (1 mM), and MgCl_2_ (1 mM). Glass capillaries (a 2.5-mm diameter, 7-cm length) were quickly filled with homogenate and immediately frozen in liquid nitrogen. All chemicals were purchased from Merck (former Sigma-Aldrich; St. Louis, MO, United States). Fluorescence emission spectra of *A. thaliana* homogenates were recorded using a steady-state spectrofluorimeter (Perkin Elmer LS-55B, United Kingdom) at 77 K with excitation wavelength of 440 nm and emission, ranging from 600 to 700 nm (slits – 10 nm; scan speed, 100 nm/min; internal emission, 515 cut-off filter). After background subtraction, intensities of Pchlide fluorescence peaks at 654 and 633 nm were read from the spectra and used for the calculation of the Pchlide_654_/Pchlide_633_ ratio.

### Analysis of Pigment Composition

Weighted seedlings (about 100 mg) were lyophilized in darkness, weighed again and extracted with small portions of the extraction solution containing: acetonitrile:ethyl acetate:0.2-M ammonium acetate (8:1:1, v/v/v). Aliquots of 100 μl of pigment extracts were separated using HPLC (PU-2089 Plus, JASCO), coupled with a UV-VIS detector (MD-2015 Plus, JASCO), using an octadecasilane column (Tracer Excel 120 ODSA 5 μm 25 × 0.4 cm, Teknochroma). The elution protocol is given in Supplementary Data 2. HPLC grade solvents were purchased from Chempur (Piekary Slaskie, Poland). Pigment identification was carried out according to the elution sequence (Wright et al., 1991, 2006) and UV-VIS absorption spectra measured between 270 and 700 nm (±1.5 nm) (Baumeler and Eugster, 1992; Baumeler et al., 1994; Egeland et al., 2011; Mysliwa-Kurdziel et al., 2012). Amount of each xanthophyll was determined based on the maximum absorbance peak (between 425 and 460 nm) area and normalized to seedling dry weight (DW).

### Antioxidant Determination Using the EPR Method

Antioxidants were extracted from weighted etiolated seedlings (70–100 mg) in 2 ml of ice-cold methanol (>99.9%, Chempur). Methanolic extracts of etiolated seedlings were mixed evenly by volume with freshly prepared 1 mM ice-cold methanolic solution of 1,1-diphenyl-2-picrylhydrazyl (DPPH, Sigma-Aldrich). The spectra were measured at 20°C, using a MiniScope MS300 (Magnettech GmbH, Germany) spectrometer with a frequency of 9.4 GHz, amplitude modulation of 1,700 mG, microwave power of 10 mW, and a scan rate of 280 G/min. First measurement was taken immediately after sample preparation, the second one after 30 min of dark incubation at room temperature. Scavenging of DPPH was calculated to quercetin (>95%, HPLC grade, Sigma-Aldrich) equivalent [according to Pervin et al. (2016)] and normalized to seedling FW.

### RNA Isolation and qRT-PCR

About 50–100 mg of cotyledons was frozen in liquid nitrogen and grounded to powder. About 1 μg of total RNA extracted with Spectrum™ Plant Total RNA Kit (Sigma-Aldrich) was reverse transcribed using RevertAid First Strand cDNA Synthesis Kit and oligo(dt)18 primers. Real-time reverse transcription (qRT-PCR) was performed using SYBR Green JumpStart Taq ReadyMix (Sigma Aldrich), 0.5 μM of primers (except *SAG12* and *SAG13* for which 0.33 μM was used) and amount of cDNA corresponding to 50 ng of RNA.

Primer sequences were: *PORA* (for: 5′-AGAGTCTAGTCT GTTCGGTGTTTCAC-3′; rev: 5′-CTGATGGAGTTGAAGTCG CGATTGC-3′) *PORB* (for: 5′-CCGACCAAATCAAATCCGAA CATGGA-3′; rev: 5′-GTGGCTAGACCTAACCCAGACGAG-3′) or *PORC* (for: 5′-AGATAAGCGTTGGAACCAACCATCTC-3′; rev: 5′-AACTGTTTTGCCCATTCAATCCTGAC-3′), reference genes (Czechowski et al., 2005) – *PDF* (for: 5′-TAACGTGG CCAAAATGATGC-3′; rev: 5′-GTTCTCCACAACCGCTTGGT-3′), *SAND* (for: 5′-AACTCTATGCAGCATTTGATCCACT-3′; rev: 5′-TGATTGCATATCTTTATCGCCATC-3′) and *UBC* (for: 5′-CTGCGACTCAGGGAATCTTCTAA-3′; rev: 5′-TTGTG CCATTGAATTGAACCC-3′), senescence markers (Chou et al., 2018): *SAG12* (for: 5′-TGCAGTAACTGCGATTGGATAC-3′; rev: 5′-TTGATGATCCAATACTTTGATC-3′) and *SAG13* (for: 5′-ATTTAGATGTGTCCACATGTTC-3′; rev: 5′-CCACGCAAGCATAAATATCTAA-3′). The PCR reactions were performed using Illumina Eco™ Real-Time PCR System with Eco Control software (Eco Real-Time PCR System). Reactions were initiated at 95°C for 10 min, followed by 40 cycles of 15 s at 95°C, 15 s of annealing (53°C for *PDF*, 55°C for *PORA*, or 56°C for other primers) and 20 s at 72°C. GeNorm v3.4 (Vandesompele et al., 2002) was used to calculate the normalization factor for all reference genes (PDF, SAND, and UBC). The normalization factor was then used to calculate the expression levels of the *POR* genes using the ΔΔC_*t*_ method (Livak and Schmittgen, 2001). All the results were normalized to the expression levels of the WT seedling after 4 days of etiolation. The modified ΔΔC_t_ method, using *LPORB* as a reference, was used to calculate *LPORA:LPORB* and *LPORC:LPORB* ratios.

### Ion Leakage Analysis

Weighted seedlings (about 100 mg) were immersed with distilled water (2 ml) and incubated in darkness at room temperature for 90 min, followed by measurement of liquid conductivity (A) using Econo™ Gradient Monitor (Bio-Rad). Additionally, the conductivity of boiled (15 min at 100°C) samples was measured to estimate the maximal ion leakage (B). The index of injury was calculated using (100% × A)/B equation based on (Flint et al., 1967).

### Acquisition of Photographic Images of Seedlings

Seedlings on culture plates were photographed immediately after removing the aluminum foil covering the plates. The images were then used for analysis of seedling morphology concerning the shape of cotyledons and apical hook curvature. A stereo microscope (SK Series Microscope, OPTA-TECH, Poland) equipped with an HDMI series camera and OPTAView-IS (ver. 4.3.0.6001) software was used for image acquisition and processing. Seedling lengths were calculated using ImageJ v1.52a (Schneider et al., 2012).

### Statistical Analysis

All biochemical analyses, described in sections “Carotenoid and Pchlide determination” to “Ion leakage analysis”, were performed for 3–4 independent experiments, consisting of pooled seedling samples (the amount of seedlings is indicated in each section above). Absorption, fluorescence and EPR spectra, chromatograms, and PCR reactions were performed in triplicate. Seedling morphology was estimated using 100–300 seedlings per experiment, and the experiment was repeated three to five times. Data for plot representation were calculated as an arithmetic mean with a standard deviation (SD). Results were analyzed *via* two-way ANOVA for the effect of mutation and the length of the etiolation, and *post hoc* Tukey’s test. Differences were considered statistically significant for *p* < 0.05. For comparison of seedling morphology, Kruskal-Wallis with *post hoc* Dunn tests was used.

## Results and Discussion

### Seedling Growth and Morphology

Although the scale of seedling development according to Boyes et al. (2001) is widely used to design experiments using light-grown *A. thaliana*, it is not applicable for dark-grown seedlings, and no such scale has been developed for etiolated plants. Despite the vital role of *A. thaliana* in understanding the molecular mechanisms controlling plant etiolation, data on morphology of etiolated *A*. *thaliana* seedlings at different age are scarce. On the contrary, numerous manuscripts for cereals, *Beta vulgaris*, *Phaseolus vulgaris*, or *Pisum sativum* (Boffey et al., 1980; Schoefs and Franck, 1993; Amrani et al., 1994; He et al., 1994; Niroula et al., 2021) can be found. Moreover, protocols used to induce etiolation differ among reports, among others, in stratification and growth conditions, light pretreatment, as well as in addition of sugar and its concentration in the culture medium.

In the present study, etiolated *A. thaliana* seedlings (WT and *lut2* mutant) were grown aseptically for different times between 2 and 12 days, counted from the end of stratification. The average fresh weight (FW) of seedlings collected at subsequent days of growth in darkness is shown in Table 1. An increase of FW was slightly faster for WT seedlings compared to the *lut2* mutant up to the 8^th^ day of growth. The FW of WT seedlings reached the maximum at day 6 and then decreased, while that of *lut2* seedlings continued to grow till the end of analysis. The difference between the repetitions of the same experiment was *ca.* 20% on average. The decrease in FW observed for WT at the end of the dark cultivation period (i.e., 8, 12 days) is likely due to water loss, which does not appear to be the case for the *lut2* mutant. Further investigation into water status of etiolated seedlings is required to explain this observation. However, this interesting observation may be easily overlooked in typical experiments aimed at comparison of seedlings, including various mutants, only at a certain etiolation day. On the other hand, FW is often used as a normalization factor. Our results show that such approach to biochemical analysis may involve errors resulting from the loss of seedling weight during etiolation. Data shown in Table 1 are useful for optimizing the consumption of seeds and planning experiments, which are required to obtain intended seedling biomass after a given period of seedling growth.

**TABLE 1:** The fresh weight (FW) of seedlings normalized to 1 mg of seeds that were used for the culture.

Time of etiolation (days)	WT (mg)	*lut2* (mg)
2	6.2 ± 1.6	5.1 ± 0.3
3	12.3 ± 2.7	9.4 ± 1.1
4	19.5 ± 1.7	14.3 ± 4.7
6	26.0 ± 4.9	20.9 ± 5.8
8	24.7 ± 4.9	23.0 ± 3.9
12	17.0 ± 4.8	30.7 ± 5.4

*The given values are averages and SD of three independent repetitions of the experiment; n = 3. The time of etiolation is counted, starting from the end of stratification. The germination ratio did not differ between lut2 and WT.*

**FIGURE 1:**
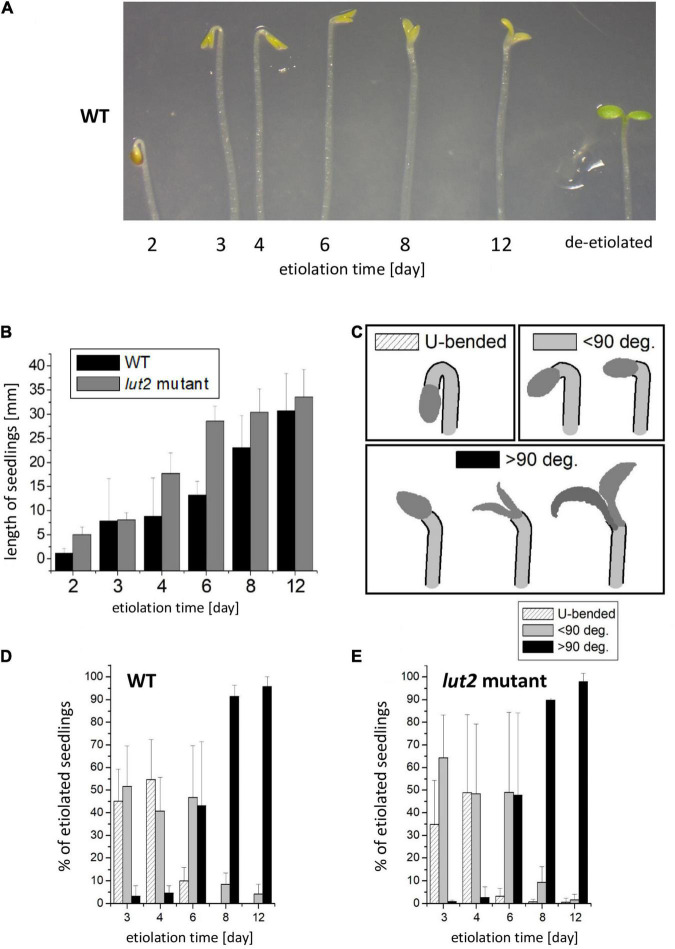
Morphology of dark-grown *Arabidopsis thaliana* seedlings of wild type (WT) and *lut2* mutant. **(A)** A representative picture of seedlings showing the cotyledon opening and the curvature of an apical hook during the etiolation; for a comparison, 6-day-old etiolated seedling then de-etiolated for 24 h is shown. **(B)** The length of seedlings. Bar heights represent arithmetic mean, while error bars represent SD; *n* = 3 repetitions (100–300 seedlings per replicate). **(C)** Representation of arbitrary categories for seedling classification regarding the curvature of an apical hook. **(D,E)** The percentage share of the particular categories in the population of seedlings at different ages for WT and *lut2* mutant. Bar heights represent arithmetic mean, while error bars represent SD; *n* = 5 repetitions (100–300 seedlings per replicate). Statistically significant differences for data shown in panels **(C–E)** are shown in Supplementary Data 5.

Results of the analysis of seedling morphology are presented in Figure 1. We have observed some differences among seedlings of the same age concerning the shape of the apex. In each culture, there were seedlings having a U-bended apical hook. However, one could also find seedlings whose apical hook was not bent, and those having additionally partly opened cotyledons, mostly after day 6. Examples of the observed apical hooks and cotyledon shapes, including atypical shapes for etiolated seedlings are shown in Figure 1A (see also Supplementary Data 3). Emerging seedlings could be observed as soon as at the 2^nd^ day of growth. At that stage, their growth was intense, and their average length almost doubled between 2^nd^ and 4^th^ days of development, quickly exceeding 10 mm in length (Figure 1B). The kinetic of hypocotyl growth differed between WT and *lut2* mutant, being slightly faster for the latter plants. At the day 12, both WT and *lut2* seedlings on average reached about 30 mm. To analyze differences in the curvature of an apical hook and cotyledon opening among seedlings in the same culture, and to quantify them, we arbitrarily classified seedlings into three categories according to the shape of the apical hook and the cotyledon expansion (Figure 1C). The first category included seedlings with a U-bended apical hook and closed cotyledons (Figure 1C; U-bended), which were observed predominantly during the first 4 days of growth in darkness (Figures 1D,E). In the second category, we included seedlings with the hook curvature equal or less than 90° (Figure 1C; <90). These were mostly observed during the first 6 days of growth (Figures 1D,E). Seedlings with only a slightly bended apical hook were included to the third category (Figure 1C; >90). Typically, 2/3 of these seedlings had also partially opened cotyledones. Seedlings from the third category were mostly observed after day 8 (Figures 1D,E). No significant differences were found between WT and *lut2* mutant; thus, the lack of lutein does not affect hook formation and maintenance. Importantly, for 6-day-old seedlings and younger, which are usually taken for de-etiolation study, the straightening of the apical hook and the opening of cotyledons were rarely observed (Figures 1D,E). However, even 12-day-old seedlings, mostly belonging to the third category, retain the ability to undergo de-etiolation (not shown).

At first glance, the straightening of the apical hook and the opening of cotyledons seem surprising in dark-grown seedlings. These processes are considered as characteristics for the initial seedling de-etiolation. Notably, in our experiments, the seedlings were carefully protected from light since their stratification until their harvest under scattered dim green light that was confirmed to be inactive in the de-etiolation process. Additionally, at various stages of the research, we performed control tests to check whether the seedlings were accidentally illuminated (Supplementary Data 4). We did not observe any signs of accidental seedling illumination in the described experiments. The formation of the apical hook is commonly regarded as the canonical effect of etiolation. This process in *A*. *thaliana* depends on auxin activity, modulated by the cooperation between ethylene, brassinosteroids, and gibberellins (Vriezen et al., 2004; Vandenbussche et al., 2010; Gallego-Bartolomé et al., 2011; Smet et al., 2014; van de Poel et al., 2015). At the molecular level, the cooperation of PIFs (Phytochrome Interacting Factors) and COP/DET/FUS proteins is essential for evoking the etiolated phenotype, including hook formation (Hardtke and Deng, 2000; Bauer et al., 2004; Leivar et al., 2008). However, the analysis of pictures described as “representative-etiolated seedlings” in numerous already published articles [for examples (Rodríguez-Villalón et al., 2009b; Xue et al., 2012; Chen et al., 2013; Kong et al., 2015)] revealed that the shape of the apex in etiolated seedlings can be very diverse, similar to our observations. This phenomenon seems overlooked and usually not commented. In our work, we categorized the observed shapes of the apex and presented quantitatively how their distribution changed in the course of seedling growth in darkness (Figures 1D,E).

Temperature affected the growth of etiolated seedlings of *A*. *thaliana* and seemed to have an impact on hook curvature (Kong et al., 2015). De-etiolation features occurring in dark have already been well documented for *A*. *thaliana* seedlings that were grown in the presence of exogenous sugar and irradiated after stratification (Žádníkova et al., 2010). Both, the presence of sugar in a culture medium and the light pretreatment applied after the stratification, should be regarded as factors that can influence the shape of the apex in etiolated seedlings. The effect of sugars on various physiological processes in *A. thaliana* has already been shown, including cell signaling pathways (Dijkwel et al., 1997), activities of phytohormones (Price et al., 2003; Loreti et al., 2008; Li et al., 2014), and photosynthesis (Eckstein et al., 2012). Glucose and mannose have been reported to delay germination (Pego et al., 1999; Dekkers et al., 2004). Elevated concentration of exogenous glucose was indicated as an inductor of post-germination developmental arrest (Rolland et al., 2002), and sucrose was shown to elevate the anthocyanin content in dark-grown seedlings (Teng et al., 2005; Li et al., 2014). In etiolated seedlings grown in the presence of sucrose, an increase of the PLB size and loosening of its structure accompanied with changes in Pchlide biosynthesis were observed (Bykowski et al., 2020). To avoid any sugar effects, we used no exogenous sugar in the culture medium, which also serves a better model mimicking natural conditions.

Light pretreatment, which is widely used in research on dark-grown *A. thaliana*, stimulates germination in darkness *via* activating the initial pool of phytochrome B (PHYB) (Shi et al., 2013). We have previously shown that the pretreatment even with dim white light (<10 μmol photons m^–2^ s^–1^) applied before the stratification substantially improved germination (Jedynak et al., 2013). In our experiments, the light pretreatment proceeded stratification. However, in some studies, the light pretreatment followed the stratification [for example in Žádníkova et al. (2010) and Gallego-Bartolomé et al. (2011)]. It needs to be taken into account that illumination of imbibed seeds after stratification activates the pool of accumulated PHYB, and, to some extent, also – PHYA, which alters physiological responses and counteracts the etiolation process driven by PIFs activity. Thus, the light treatment after stratification is often considered as pseudo-dark conditions (Josse and Halliday, 2008; Leivar et al., 2008; Galvão et al., 2012). The straightening of the apical hook and the opening of cotyledons in dark-grown seedlings were already observed under pseudo-dark conditions (Žádníkova et al., 2010; Gallego-Bartolomé et al., 2011). These processes started after 50 h after germination in darkness and finished after 120 h (6 days). In our study, these phenomena occurred also under true dark conditions, i.e., without irradiation of seeds after stratification, although they were slower compared to pseudo-dark conditions and observed mostly after day 6 both for WT and the *lut2* mutant (Figures 1D,E).

Our results clearly demonstrated that seedling population grown in the absence of light is heterogeneous, and assumption that etiolated seedlings are identical concerning the appearance of the apex is an oversimplification. The etiolation of *A. thaliana* should be perceived as a dynamic process, and growth protocols need to be taken into account when comparing results for etiolated seedlings. We advocate the opinion that etiolation methodology used for *A. thaliana* research should be standardized to enable comparison of results, and our work may serve as the reference. Moreover, any modifications, for example, necessary to study mutants should be clearly reported.

### Stress and Senescence

One may think that etiolation for up to 12 days is a highly unfavorable and detrimental phenomenon. To verify whether the observed straightening of the apical hook and the opening of cotyledons (Figures 1D,E) are related to stress or senescence, we checked the total antioxidant activity, ion leakage, and the expression of particular senescence markers.

**FIGURE 2:**
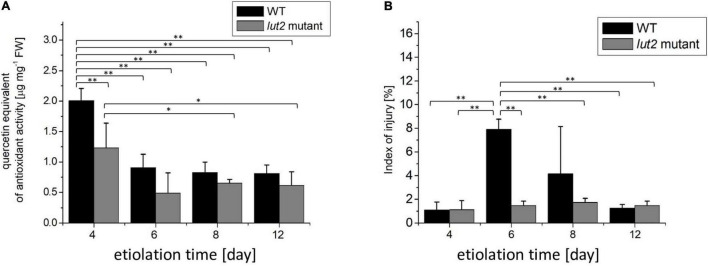
Total antioxidant activity and ion leakage in etiolated WT and *lut2* mutant. **(A)** Total antioxidant activity of seedling extracts based on the EPR signal of DPPH expressed as quercetin equivalent. **(B)** The index of injury expressed as percentage of ion leakage at certain condition to maximum ion leakage. Bar heights represent arithmetic mean, while error bars represent SD, *n* = 4. Statistically significant differences from two-way ANOVA and *post hoc* Tukey test were indicated using ^**^p < 0.01; *p < 0.05.

Stress or senescence may decrease the content of antioxidants. DPPH is a stable radical commonly used for determination of antioxidant capacity of plants (Moon and Shibamoto, 2009). The total antioxidant activity assessed with DPPH is contributed to plant ascorbic acid (Krishnan et al., 2015) as well as to phenolic and flavonoid compounds (Ashafa et al., 2010), which all are abundant in *A*. *thaliana* (Fraser and Chapple, 2011). The total antioxidant activity significantly decreases in severely stressed seedlings (Lakshmi Sahitya et al., 2018) or during senescence (Liang et al., 2018). In our study, the highest antioxidant level was detected in 4-day-old seedlings (Figure 2A), and it decreased with increasing the etiolation time. The antioxidant content was slightly lower in *lut2* than in WT seedlings, although similar tendencies were observed in both cases. Having the highest antioxidant content, relatively young (4-day-old) seedlings seem to be protected from oxidative stresses better than the older ones. The observed decrease in antioxidant content indicates that long etiolation is somehow related with stress. However, it needs to be noted that the antioxidant pool seems not to be significantly depleted even in 12-day-old etiolated seedlings.

The increased ion leakage has been associated with the increased cell membrane permeability and cell damage observed for severe stresses or senescence (Demidchik et al., 2014; Chen et al., 2016). An index of injury is a relative ion leakage under certain conditions to a maximum ion leakage, and a useful parameter to assess plant vitality. In unstressed *A*. *thaliana*, it does not exceed 20% and typically fluctuates around 10% (Zhang et al., 2004; Liu et al., 2015). In stressed plants, the index of injury is much higher and usually ranges from 30 up 90% (Zhang et al., 2004; Liu et al., 2015; Wang et al., 2016; Ilík et al., 2018; Jiang et al., 2021). In our study, values of the index of injury were lower than 10% (Figure 2B), indicating no stress or cell damage of seedlings grown in darkness for up to 12 days. However, a transient increase of this parameter was noted for 6- and 8-day-old WT seedlings. Even though, the maximal values stay below 10%, thus not pointing to stress or cell damage.

We also compared the expression of senescence-associated genes *SAG12* and *SAG13* for 4- and 12-day-old seedlings. *SAG* genes are considered as molecular markers of senescence. Particularly, *SAG12* is a very specific marker of age-related senescence, while *SAG13* plays a role in senescence induced by natural aging as well as may be induced by oxidative stress and pathogens. The *SAG13* transcript level strongly increases also in individually darkened green leaves (Weaver and Amasino, 2001; Schippers et al., 2007; Chen et al., 2017; Dhar et al., 2020). *SAG13* transcripts are considered as early markers of senescence, as accumulation of these transcripts precedes expression of other senescence-associated genes, including *SAG12* (Schippers et al., 2007). Irrespective of seedling age, *SAG12* and *SAG13* transcripts were hardly detected in etiolated seedlings - C_t_ values were higher than 30 (not shown). Moreover, any significant age-related increase in content of *SAG12* and *SAG13* mRNA transcripts has not been observed, which may indicate that darkness does not induce senescence in seedlings under our experimental conditions.

To sum up, our results clearly showed that *A. thaliana* seedlings, even at day 12 of etiolation, did not show obvious symptoms of neither stress nor senescence and seemed healthy.

### Pigment Accumulation

**FIGURE 3:**
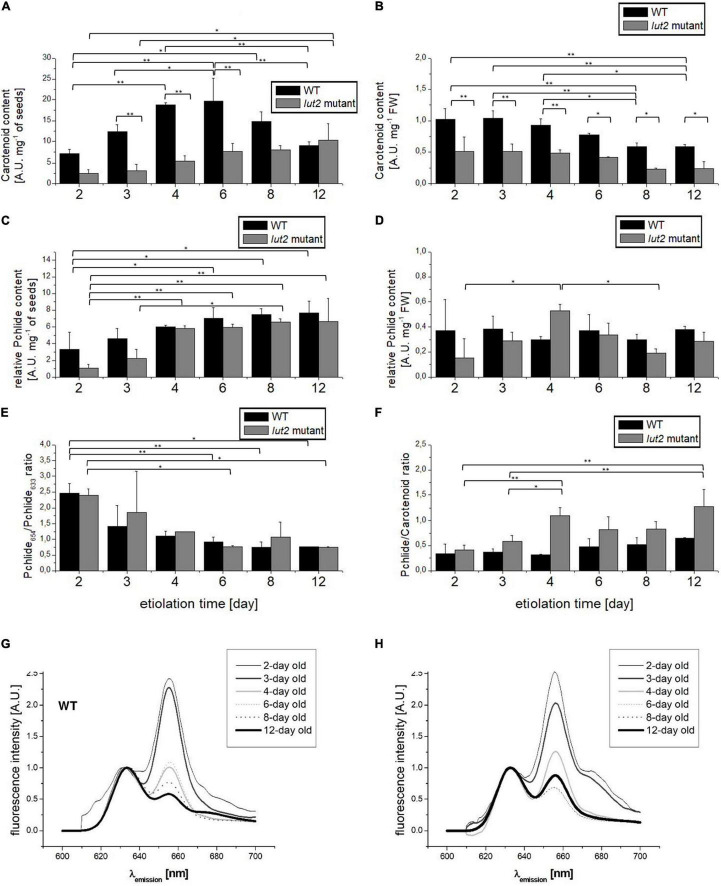
Analysis of pigments in etiolated seedlings. **(A,B)** Total carotenoid content normalized to seed weight or to seedling FW. **(C,D)** Total Pchlide content estimated fluorometrically and normalized to seedling weight or to fresh weight of seedlings. **(E)** The Pchlide_654_/Pchlide_633_ ratio measured at 77K. **(F)** The ratio between total Pchlide and carotenoid content. **(G,H)** Representative comparison of Pchlide emission spectra (λ_exc_ = 440 nm) from etiolated seedlings of WT and *lut2* mutant at different ages, measured at 77K and normalized at 633 nm. Bar heights represent arithmetic mean, while error bars represent SD; *n* = 4. Statistically significant differences from two-way ANOVA and *post hoc* Tukey test were indicated using ^**^p < 0.01 and *p < 0.05.

During the analysis of pigment content, i.e., carotenoids and Pchlide, we faced the problem of result normalization. A commonly used normalization by FW may be improper because of high water content in etiolated seedlings, which may differ for WT and *lut2* mutant, as it was discussed in section “Seedling Growth and Morphology.” Taking into account that we have not observed differences concerning the seed weight and the germination ratio between the *lut2* mutant and WT (not shown), the initial mass of seeds that were sown well correlated with the number of seeds, and, in consequence, with the number of seedlings. Thus, for comparison, we performed both the normalization of the pigment content by the mass of sown seeds (Figures 3A,C) and per seedling FW (Figures 3B,D).

Regardless of the way of normalization, WT accumulated more carotenoids than *lut2* mutant within the whole investigated etiolation period (Figures 3A,B). As lutein is one of the most abundant carotenoids accumulated in darkness (Mysliwa-Kurdziel et al., 2012), thus the reduction of carotenoid content in *lut2* mutant may be explained as a direct result of the lack of ε-lycopene cyclase encoded by *LUT2* and impaired biosynthesis of this pigment (Park et al., 2002). When normalized by FW (Figure 3B), the highest content of carotenoids was observed in the youngest seedlings of both WT and the *lut2* mutant, and it gradually decreased with the seedling age. It can be partially attributed to a rapid increase in the mass of seedlings (Table 1). Water accumulation is the most probable reason of the mass increase, as due to the lack of photosynthesis in darkness and lack of sugar in culture medium biomass accumulation is unlikely. The effect of normalization was so profound that small differences in carotenoid content were undetectable with that approach. The variations in carotenoid contents became visible when they were normalized by the seed mass (Figure 3A). In this case, the carotenoid content in WT increased between 2^nd^ and 6^th^ days of growth in darkness and then lowered in the older seedlings. On the contrary, slight but constant increase in average carotenoid content was observed for the *lut2* mutant. Thus, it seems that lack of lutein influences the total carotenoid accumulation. It might be possible that lutein is important for the control of the overall carotenoid content in etiolated seedlings, influencing this way the kinetics of accumulation of these pigments.

We used fluorometric method for semi-quantitative estimation of Pchlide content and normalized the results by FW or by seed mass. High sensitivity of the fluorescence method allowed us to detect the initial pool of Pchlide as early as in 2^nd^ day of etiolation (Figures 3C,D). When the results were normalized by the seed mass, the slight and gradual increase in Pchlide content during subsequent days of etiolation was observed (Figure 3C). In 2-day-old WT seedlings, it was significantly lower than in older seedlings. In *lut2* mutant, the Pchlide content was significantly lower in 2- and 3-day-old seedlings. At the beginning of growth, Pchlide accumulation seems slower in the mutant than in WT seedlings. No changes in Pchlide content nor significant differences between *lut2* mutant and WT seedlings were observed when data were normalized by FW (Figure 3D). These results show that Pchlide accumulation was balanced with increasing weight of seedlings. However, it is worth to note that, whereas the average Pchlide content in WT seedlings was comparable during the whole experiment, in *lut2* mutant, it reached the maximum at the 4^th^ day of growth. It seems that Pchlide accumulation is slightly slower in *lut2* mutant. On the other hand, it cannot be excluded that lutein may play a slight but noticeable role in regulation of Pchlide biosynthesis in dark.

We calculated the ratio of Pchlide to carotenoids content to verify if the accumulation of these pigments is coupled and depends on lutein (Figure 3F). At the early growth stage (the day 2), the Pchlide/carotenoid ratio was similar in WT and *lut2* mutants, but, in the older seedlings, the average values were higher for the mutant, especially in 3- and 4-day-old seedlings. These results point to different kinetics of Pchlide and carotenoid accumulation in the *lut2* mutant as compared with WT (Figures 3A,C,F). Notably, 6- and 8-day-old seedlings of *lut2* exhibited no significant differences in comparison to WT, suggesting that, at this stage, the biosynthesis of carotenoids and Pchlide are, at least to some extent, co-regulated and balanced, even in the absence of one of the most abundant xanthophylls – lutein.

Physiological state of the accumulated Pchlide can be revealed by the analysis of fluorescence emission spectra measured at 77 K (Belyaeva and Litvin, 2007; Solymosi and Mysliwa-Kurdziel, 2021). Two fluorescence bands can be easily distinguished with emission maxima at about 633 and 654 nm, similar for WT and *lut2* mutant (Figures 3G,H). These bands have already been attributed to specific Pchlide forms that were characterized at the biochemical level (Sironval and Brouers, 1970; Böddi et al., 1992; Mysliwa-Kurdziel et al., 2003; Schoefs and Franck, 2003). The band at 654-nm origins from the photoactive Pchlide (Pchlide_654_), i.e., Pchlide bound to the active site of LPOR and forming ternary Pchlide: LPOR: NADPH complexes in PLBs, which are ready for Pchlide to Chlide reduction upon illumination (see Supplementary Data 4). The other band (with the maximum at 633 nm) represents a non-photoactive Pchlide (Pchlide_633_), which is not converted to Chlide with a pulse of light. In our study, the relative amount of the photoactive Pchlide, calculated as the Pchlide_654_/Pchlide_633_ ratio, decreased with the seedling age (Figures 3E–H), mostly between the 2^nd^ and 3^rd^ days. Then, the changes in this ratio were subtle, and the lowest value (about 0.75) was observed after day 8. No significant differences between *lut2* and WT were observed. That is in contrast to results obtained for *Phaseolus vulgaris*, where the low Pchlide_657_/Pchlide_632_ ratio was observed in 2-day-old seedlings and high in 10-day-old seedlings (Schoefs and Franck, 1993). In etiolated wheat seedlings – the relative Pchlide_654_ content depended on the seedling age along coleoptiles (Solymosi and Aronsson, 2013; Leonowicz et al., 2018). Thus, kinetics of formation of photoactive complexes is not universal and depends on the species.

**FIGURE 4:**
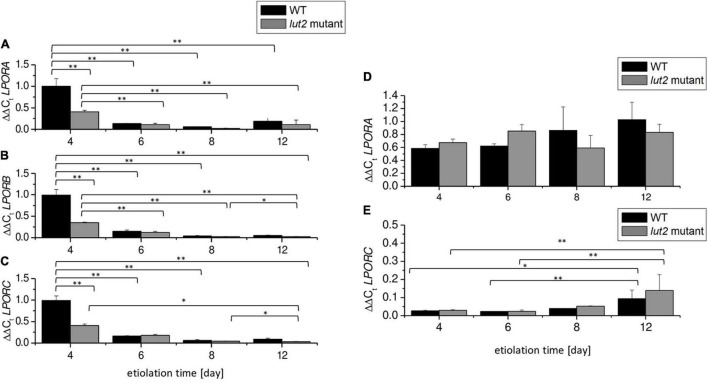
Accumulation of *LPOR* transcripts. **(A–C)** ΔΔC_t_ values of *LPORA*, *LPORB*, and *LPORC* genes using the normalization factor obtained *via* GeNorm v3.4 from *PDF*, *UBC*, and *SAND* reference genes. **(D,E)** Re-calculation of expression of *LPORA* and *LPORC* in relation to the content of mRNA-encoding *LPORB* at a certain day of etiolation in WT and *lut2 mutant*. Bar heights represent arithmetic mean, while error bars represent SD; *n* = 3. Statistically significant differences from two-way ANOVA and *post hoc* Tukey test were indicated using ^**^p < 0.01 and *p < 0.05.

Pchlide_654_ formation also depends on LPOR availability (Sperling et al., 1998; Gabruk et al., 2015). We checked the accumulation of *LPOR* transcripts *via* qRT-PCR with primers specific for *LPORA*, *LPORB*, and *LPORC*. We detected transcripts of all *LPORs* in etiolated seedlings, both in WT and in *lut2* mutant (Figures 4A–C). According to our knowledge, *LPORC* transcripts have not been described in etiolated seedlings so far. Northern blot analysis has already shown that etiolated *A. thaliana* seedlings contain *LPORA* and *LPORB* mRNA, with the *LPORA* to *LPORB* ratio relatively constant, whereas *LPORC* was not detected (Armstrong et al., 1995; Su et al., 2001; Masuda et al., 2003; Masuda and Takamiya, 2004). Here, we used the qRT-PCR method, which is more sensitive than Northern blotting, and allowed us to detect *LPORC* transcripts. Interestingly, changes of relative content of mRNAs of each *LPOR* followed a similar pattern. The steady-state levels of all *LPOR* transcripts were the highest in the 4-day-old seedlings and slightly lower for the *lut2* mutant compared to WT. In case of older etiolated seedlings, no significant differences between the *lut2* mutant and WT seedlings were detected. The observed decrease of mRNA level of all the *LPORs* during the etiolation process is surprising because only the presence of light and light intensity has been mentioned so far as regulators of the accumulation of *LPORs* transcripts.

To compare the expression of *LPORs*, we recalculated the levels of *LPORA* and *LPORC* using *LPORB* as a reference gene (Figures 4D,E). As a result, we were able to estimate the *LPORA (LPORC):LPORB* transcript ratios. The average mRNA levels of *LPORA* were only slightly lower than *LPORB* ones (Figure 4D). On the contrary, the amounts of *LPORC* transcripts were more than 30-fold lower than *LPORB* transcripts in 4- and 6-day-old seedlings, both in WT and *lut2* mutant (Figure 4E). This points to the potential co-regulation and balance among different *LPOR* expressions during the etiolation process.

The observed decrease in levels of *LPOR* mRNAs (Figure 4) during growth of etiolated seedlings coincided with the observed decrease in the Pchlide_654_/Pchlide_633_ ratio (Figure 3E). At the same time, the increase in Pchlide content was observed (Figures 3C,D). These results indicate that the LPOR level is too low to continue formation of Pchlide:LPOR:NADPH complexes. Some correlation between the Pchlide_654_/Pchlide_633_ ratio and LPORA or LPORB accumulation has already been proved (Sperling et al., 1998). Lutein is not crucial for the assembly of Pchlide: LPOR:NADPH complexes. Nevertheless, already suggested impact of lutein on Pchlide accumulation (Mysliwa-Kurdziel et al., 2012) should be noted (Figures 3C,D).

Our results prove that, in etiolated seedlings of *A*. *thaliana*, the accumulation of *LPOR* transcripts and Pchlide is uncoupled. Insufficient LPOR levels result in the increase of the relative amount of non-photoactive Pchlide (Pchlide_633_), visible in fluorescence spectra (Figures 3G,H). In the presence of light, the Pchlide_633_ pool may act as a photosensitizer inducing ROS production, especially ^1^O_2_ (Erdei et al., 2005). The overaccumulation of LPOR proteins (resulting in the increase of the Pchlide_654_/Pchlide_633_ ratio) counterbalances this risk as decreased mortality of seedlings during their de-etiolation is observed (Sperling et al., 1997, 1998; Op Den Camp et al., 2003). Considering that fact, our results suggest the etiolation of germinating *A. thaliana* seedlings should not extend over 6 days, and prolonged growth in dark rapidly increases the susceptibility of seedlings to death. This needs to be considered during planning experiments using etiolated *A. thaliana* seedlings.

It is already known that Pchlide biosynthesis is partially inhibited by a negative feedback loop orchestrated by FLU (FLUorescent in blue light), the repressor of glutamyl-tRNA reductase (Meskauskiene et al., 2001; Bang et al., 2008; Kauss et al., 2012). Glutamyl-tRNA reductase is the enzyme catalyzing the first reaction of the tetrapyrrole biosynthesis pathway. Our results showed that Pchlide accumulation slowed down significantly only after the 4^th^ day of etiolation, as revealed from data normalized by seedling amount (Figure 3C). However, this was not evident if the normalization by FW was applied (Figure 3D). Pchlide_633_ activates FLU, leading to inhibition of Pchlide biosynthesis by a negative feedback loop. Interestingly, in etiolated *lut2* seedlings, the expression of *LPOR*s was significantly lowered at 4^th^ day. Young *lut2* seedlings accumulated lower amount of Pchlide than WT. That might be explained by strong inhibition of Pchlide biosynthesis and LPOR transcription at the early developmental stage. Thus, lutein may be partially involved in fine-tuning of Chl biosynthesis regulation.

### Xanthophyll Composition

The HPLC analysis allowed us to identify and quantitatively estimate the content of neoxanthin, violaxanthin, antheraxanthin, zeaxanthin, and lutein in seedlings as young as 4-day-old (Supplementary Data 2). For younger seedlings (2- and 3-day old), the xanthophyll content was below the limit of detection (LoD). Neither lutein nor zeaxanthin was detected in etiolated *lut2* seedlings irrespectively of their age. Lack of lutein is obvious for this mutant. Whereas zeaxanthin has been detected in light-grown *lut2* seedlings (Pogson et al., 1998), the lack of this carotenoid in etiolated *lut2* mutant has not been shown before. Notably, in contrast to lutein (α-carotene derivative), the zeaxanthin represents a different branch (β-carotene derivative) of the xanthophyll biosynthesis pathway (Cazzonelli and Pogson, 2010). Moreover, zeaxanthin serves as a substrate for synthesis of anthera- and violaxanthin, both carotenoids found in *lut2* mutant (Table 2). It has already been shown that, in darkness, violaxanthin was preferentially accumulated on the expanse of zeaxanthin (Pfündel and Bilger, 1994). In etiolated *Nicotiana tabacum*, the expression of zeaxanthin epoxidase seemed higher than violaxanthin de-epoxidase, favoring violaxanthin accumulation (Woitsch and Römer, 2003). A high level of violaxanthin has also been shown in etiolated seedlings of *Phaseolus vulgaris* (Schoefs et al., 1995), *A. thaliana* (Mysliwa-Kurdziel et al., 2012), and cereals (Savchenko et al., 2015). Lack of zeaxanthin observed in our study in *lut2* mutant (Table 2) indicates that the biosynthesis of violaxanthin and antheraxanthin from zeaxanthin is more efficient in etiolated seedlings of the *lut2* mutant than in WT. Thus, the presence of lutein might be crucial for maintaining a proper balance between xanthophylls and suggests that the α- and β-xanthophyll accumulation may be coordinated. Furthermore, a comparison of xanthophyll content in etiolated *lut2* and WT seedlings (Table 2) reveals that lack of lutein is not compensated by stoichiometric increase of other xanthophylls, which has been observed in light-grown mature *A. thaliana* plants (Pogson et al., 1998).

**TABLE 2:** The relative content of identified xanthophylls in etiolated seedlings of WT and *lut2* mutant.

		WT		*lut2* Mutant	
	Days in darkness	Average	SD	Average	SD
		[AU*s/mg DW]		[AU*s/mg DW]	
*trans*-Neoxanthin	4	1,554	±581	1,024^a^	±264
	6	1,329	±179	1,313^a^	±280
	8	1,296	±222	1,011	±301
	12	1,137	±310	1,198	±163
	
*cis*-Neoxanthin	4	1,443	±327	857^a^	±376
	6	1,548	±488	2,532^a^	±389
	8	1,380	±127	1,422	±984
	12	1,119	±320	922	±83
	
Violaxanthin	4	20,899^b^	±2,702	10,896^e^	±4,894
	6	15,489^abc^	±3,535	27,366^de^	±3,049
	8	11,808	±3,310	16,540	±13,323
	12	6,019^a^	±613	5,083^cd^	±718
	
Antheraxanthin	4	1,175^d^	±199	818^b^	±494
	6	824^a^	±203	2,382^abcd^	±401
	8	986^c^	±208	1,363	±733
	12	1,467	±606	1,956	±1,181
	
Zeaxanthin	4	3,258^ a^	±607	Below the LoD	
	6	1,978^ b^	±466	Below the LoD	
	8	1,840^c^	±158	Below the LoD	
	12	1,351^abc^	±327	Below the LoD	
	
Lutein	4	12,387^abc^	±1,094	Below the LoD	
	6	12,077^a^	±2,736	Below the LoD	
	8	11,926^b^	±2,808	Below the LoD	
	12	16,574^c^	±1,520	Below the LoD	

*Obtained data were normalized to dry weight (DW). trans-Neoxanthin, cis-Neoxanthin, Violaxanthin, and Antheraxanthin were analyzed via two-way ANOVA and post hoc Tukey test, while Zeaxanthin and Lutein were analyzed via one-way ANOVA and post hoc Tukey test. Statistically significant differences (p < 0.05) were indicated with the same letters. LoD – limit of detection.*

The observed kinetics of xanthophyll accumulation differed between WT and *lut2*-etiolated seedlings (Table 2). In case of WT, the content of most β-carotene derivative xanthophylls significantly and gradually decreased with seedling age. The lutein (α-carotene derivative) content was relatively stable during first 8 days and significantly higher in 12-day-old seedlings. In case of *lut2* seedlings, a transient increase of all xanthophyll content at the beginning of experiments was detected with a maximum at 6^th^ day of seedling growth in darkness.

Little is known about the physiological role of particular xanthophylls in etiolated seedlings. Carotenoid biosynthesis is regulated by phytoene synthase (PSY) – the first enzyme of the pathway. Even though, the *PSY* expression (corresponding straightforwardly to PSY activity) is strongly suppressed in etiolated *A*. *thaliana* seedlings (Rodríguez-Villalón et al., 2009b), carotenoids accumulate in darkness, which suggests their vital role. Carotenoid composition in etiolated seedlings, with lutein and violaxanthin predominantly accumulated and trace amounts of β-carotene, and neoxanthin, differs from carotenoid composition of green plants (Rodríguez-Villalón et al., 2009a). Analysis of *CRTISO A. thaliana* mutants initially pointed that lutein is crucial for the PLB assembly (Park et al., 2002; Cuttriss et al., 2007). However, further investigation assigned this effect more to the *cis*-carotenoids that accumulate in *CRTISO* mutants (Cazzonelli et al., 2020). The β-carotenoid branch led also to formation of abscisic acid (ABA). Mutants lacking ABA exhibited partially de-etiolated state, i.e., reduced hypocotyls length and increase of cotyledon expansion; they even formed true leaves in darkness (Barrero et al., 2008). Moreover, their PLB structure was locally disordered (Bykowski et al., 2020).

## Conclusions

Our results show the dynamics of the development of *A. thaliana* seedlings grown in the absence of light. They revealed that straightening of the apical hook and partial cotyledon opening occur in etiolated seedlings grown in true-dark conditions. We described heterogeneity of the morphology among seedlings in a culture. Even for a long etiolation period, seedlings do not show stress and senescence symptoms. Further research is required to elucidate molecular mechanisms of the apical hook and cotyledon opening in etiolated seedlings in true dark conditions, particularly to determine the role of hormones, PIFs factors, and COP/DET/FUS proteins.

Age-related decrease in the Pchlide_654_/Pchlide_633_ ratio, which is generally considered as disadvantageous during de-etiolation, corresponds to the age-related decrease in all *LPOR* transcripts and to Pchlide accumulation. Lack of lutein in *A. thaliana lut2* mutant has little effect on development and morphology of etiolated seedlings. However, lutein may be involved in regulation of β-xanthophylls and Pchlide biosynthesis. Further investigation is required to verify how the altered xanthophyll content and the lowered antioxidant level impact the greening of *lut2* seedlings.

Our work clearly showed that the way of normalizing results of pigment content in etiolated *A. thaliana* seedlings is very important for data interpretation. Moreover, to enable data comparison, etiolation methodology used for *A. thaliana* research should be standardized regarding the length of the etiolation time period, sugar concentration in the growth medium, and seed pretreatment with light (leading to pseudo- or true dark growth).

## Data Availability Statement

The original contributions presented in the study are included in the article/Supplementary Material, further inquiries can be directed to the corresponding author/s.

## Author Contributions

PJ and BM-K designed the experiments, analyzed the results, and worked at manuscript preparation. PJ performed the majority of experiments and coordinated the whole work. MC, PZ, and AKB planned, performed, and analyzed the qRT-PCR. KFT planned, performed, and analyzed HPLC experiments. BM-K provided funding. AKB, KFT, and PZ participated to wrote the manuscript. All authors accepted the final version of the manuscript.

## Conflict of Interest

The authors declare that the research was conducted in the absence of any commercial or financial relationships that could be construed as a potential conflict of interest.

## Publisher’s Note

All claims expressed in this article are solely those of the authors and do not necessarily represent those of their affiliated organizations, or those of the publisher, the editors and the reviewers. Any product that may be evaluated in this article, or claim that may be made by its manufacturer, is not guaranteed or endorsed by the publisher.

## Abbreviations

ABA: abscisic acid
Chl: chlorophyll
Chlide: chlorophyllide
COP/DET/FUS: constitutive photomorphogenesis/de-etiolated/fusca
DPPH: 1,1-diphenyl-2-picrylhydrazyl
DW: dry weight
EPR: electron paramagnetic resonance
FW: fresh weight
HPLC: high-performance liquid chromatography
LPOR: light-dependent protochlorophyllide: NADPH oxidoreductase
LoD: limit of detection
Pchlide: protochlorophyllide
PIF: pytochrome interacting factor
PHYA: phytochrome A
PHYB: phytochrome B
PLB: prolamellar body
PSY: phytoene synthase
qRT-PCR: real-time reverse transcription PCR
ROS: reactive oxygen species
WT: wild type *A. thaliana Columbia-*0.
