# Supplementary material for: Dynamics of Etiolation Monitored by Seedling Morphology, Carotenoid Composition, Antioxidant Level, and Photoactivity of Protochlorophyllide in Arabidopsis thaliana
Source: Front Plant Sci. 2022 Feb 22;12:772727. doi: 10.3389/fpls.2021.772727 (PMC8900029; doi:10.3389/fpls.2021.772727)
Supplement: Supplementary file 1 [file Data_Sheet_1.PDF]

Supplementary Material

1 Supplementary Data

Supplementary data S1. PCR products of plant samples which offspring was used in the study.

For genotyping of *lut2 A. thaliana* mutants the Phire Plant Direct PCR Master Mix (Thermo Fisher Scientific) kit and the standard protocol were used for the combination of 3 starters:
LP - AACAATGGTGTAAGTCTTCTCGC,
RP – TGCTAGAAGTCAGAACCTTTTCG,
SALK(LBa1) – TGGTTCACGTAGTGGGCCATCG

The expected product length for WT plants was 1100 bp, and between 457 and 757 bp for *lut2* mutants (predicted via SIGnAL T-DNA Primer Design tool).

Homozygous plants are expected to produce a single band whereas heterozygous plants should form two bands.

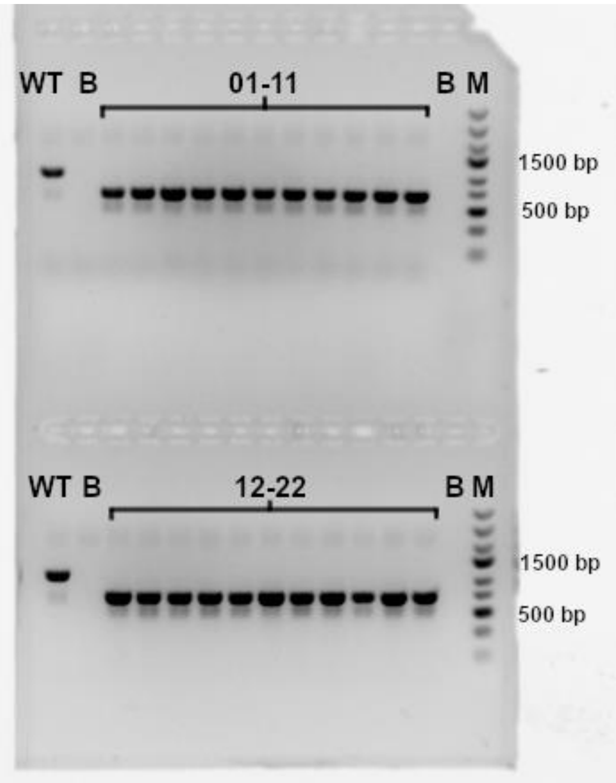

WT – *A. thaliana Col-0* sample
B – Blank sample
M – Size marker

Numbers on the gel indicate samples isolated from individual plants.
The WT plants showed a single band above the 1000 bp marker.
The *lut2* mutants showed a single band below the 750 bp marker.

Supplementary data S2. Pigment analysis - the HPLC elution and detection protocol

Weighted *A. thaliana* seedlings (about 100 mg) were lyophilised before extraction. Extraction was performed under dim green light, using the extraction solution containing: acetonitrile:ethyl acetate:0.2 M ammonium acetate (8:1:1, v/v/v). Dry-frozen samples were ground in small portions of the extraction solution until the total volume was 2 mL. Samples were centrifuged for 3 min at 5 400 rcf. The supernatant was transferred to a new centrifuge tube and centrifuged for 3 min at 14 500 rcf. Next 700 μL of the supernatant was transferred to a centrifuge filter (0.22 μm PTFE) and centrifuged once again at 10 000 rcf for 90 seconds. 100 μL of the filtrate were injected to the HPLC system. An octadecasilane column (Tracer Excel 120 ODSA 5 μm 25x0.4 cm, Teknochroma) was used for the separation. The elution program is presented in the table below.

t (min)	H_2O (%)	ACN (%)	Ethyl acetate (%)	MeOH (%)	Flow rate (mL/min)
0	5	95	0	0	0.8
19	5	95	0	0	0.8
20	0	0	50	50	1.6
26	0	0	50	50	1.6
29	5	95	0	0	1.6
30	5	95	0	0	0.8

UV-VIS (MD-2015 Plus, JASCO) and fluorescent (FP-4025, JASCO) detectors were used. The UV-VIS detector recorded the absorbance spectra between 270 and 700 nm. The peak area of maximum absorbance between 425 and 460 nm was used for quantitative measurements. Retention time and recorded spectra were used for compound identification. A representative chromatogram is presented below.

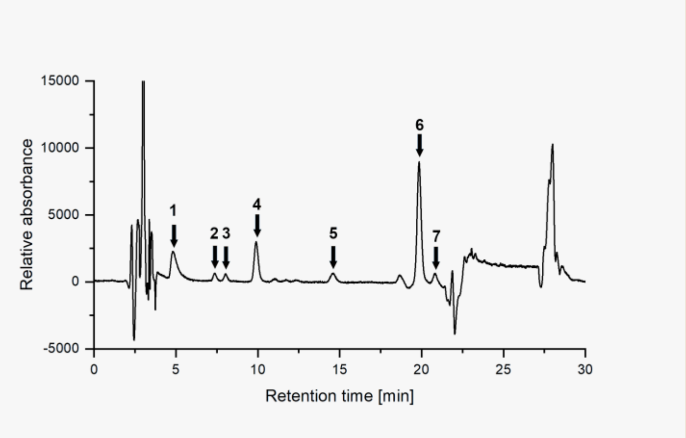

1 – Pchlide;
2, 3 – trans and cis-Neoxanthin respectively;
4 – Violaxanthin;
5 – Antheraxanthin;
6 – Lutein;
7 – Zeaxanthin.

Supplementary data S3. Heterogeneity observed among seedlings in population of the same age

Pictures of exemplary and representative seedlings taken from the population of *A. thaliana* WT seedlings at the same age (as indicated) show the heterogeneity concerning the shapes of the apical hook and cotyledons. For comparison of the hook and cotyledons shapes, seedlings that were etiolated for 6 days and then deetiolated for 24 hours are shown. Similar picture was obtain for *A. thaliana lut2* mutant.

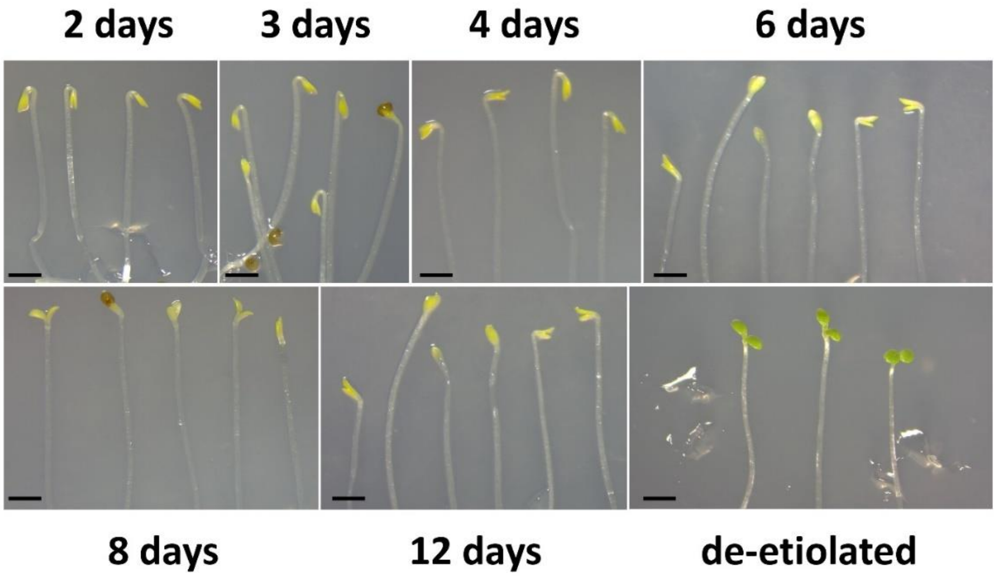

Supplementary data S4. Tests confirming etiolation of seedlings:

Several control tests described below were performed that would have allowed us to detect any incidental seedlings illumination. We did not detect any signs of induction of light - triggered processes in the case of seedlings taken for experiments.

(1) Fluorescence spectra measured at 77K for homogenates from cotyledon tissue

Fluorescence emission spectrum of homogenated cotyledon tissue was measured twice for each capillary. Capillaries were prepared as described in Material and Methods (M&M – section 2.4). The first spectrum was measured as described in the M&M – section 2.4 and described here as "fluorescence in darkness" (Figures A1 and A2). After that measurement, the capillaries with homogenate were gently warmed up (to -20°C) in darkness, then irradiated with a single flash of white light (Quantuum MOVE 200 lamp, China; energy output 200 J), and immediately frozen in liquid nitrogen. Then, the second fluorescence spectrum was measured using the same instrument setting. It is described as "fluorescence after flash of light" (Figures A1 and A2). Both spectra were normalised to 1 at 605 nm before background subtraction, then normalised to 1 at 633 nm, and used to calculate the differential "illuminated – dark" spectrum. Fluorescence band having the maximum around 688 originates from chlorophyllide, the product of the photoreduction of the photoactive protochlorophyllide. This band at 688 nm appeared only after the controlled illumination with the flash

of light together with the disappearance of fluorescence peak at around 654 nm originating from the photoactive protochlorophyllide. This spectral transformation is more evident at differential spectra.

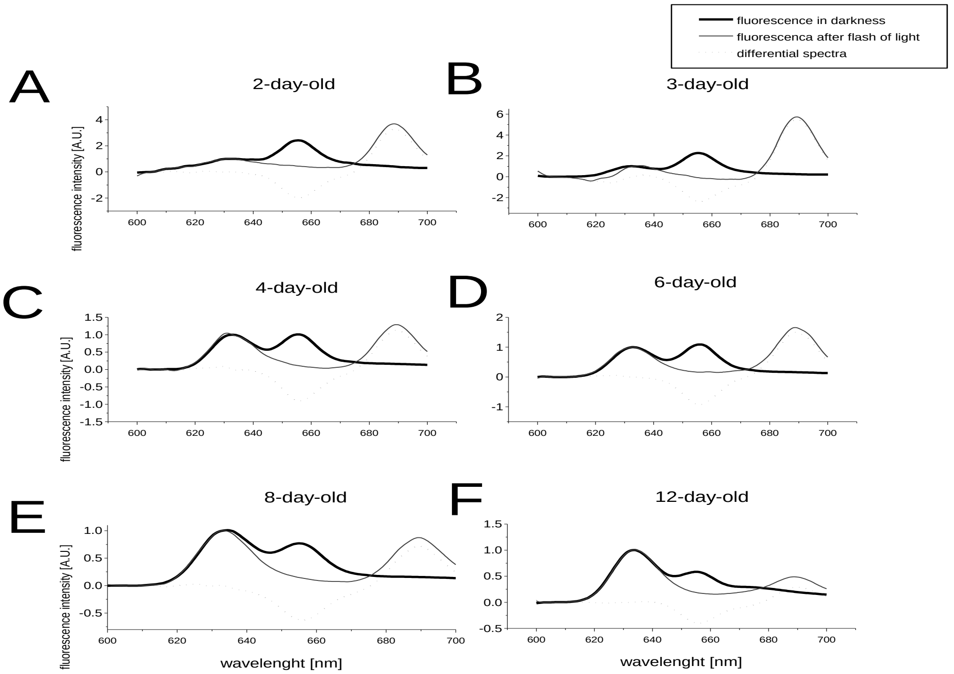

Supplementary Figure S4.1. Representative fluorescence spectra of homogenates from etiolated WT *A.* thaliana cotyledons; λ_{exc}=440 nm

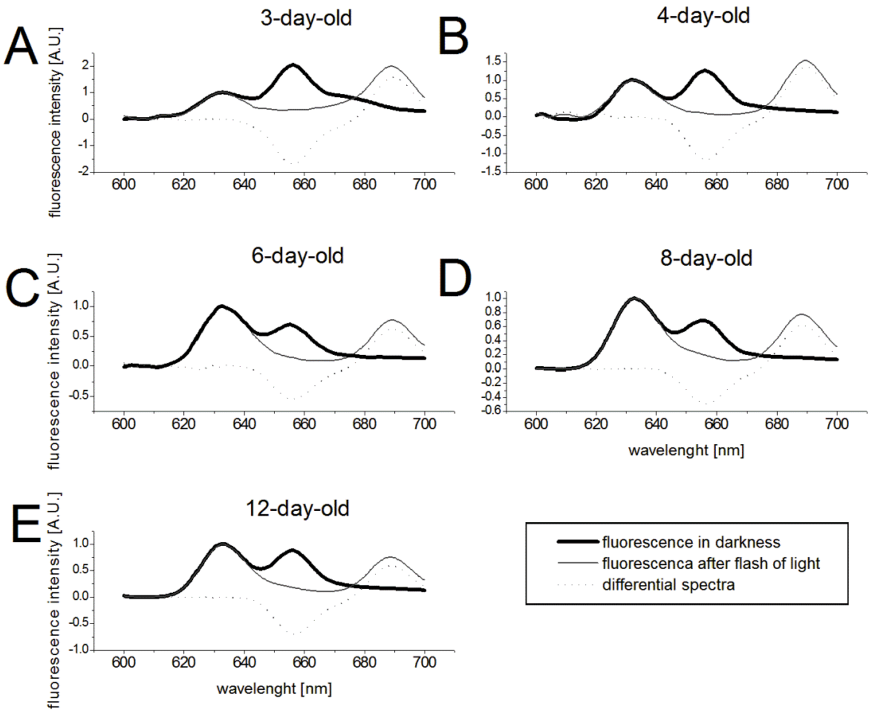

Supplementary Figure S4.2. Representative fluorescence spectra of homogenates from etiolated cotyledons of *A.thaliana lut2* mutant; λ_{exc}=440 nm

(2) Fluorescence spectra of acetone extracts measured at room temperature

Protochlorophyllide in acetone extract emits fluorescence with the maximum at 630 nm (after excitation at 440 nm) accompanied with a vibrational sub-band having maximum around 685 nm. The photoconversion of protochlorophyllide results in the formation of chlorophyllide, which emits fluorescence with the maximum at 670 nm (λ_{exc}=440 nm). The fluorescence yield of chlorophyllide is higher than that of protochlorophyllide, so trace amounts of chlorophyllide can be detected using this method. In acetone extracts, chlorophyll *a* shows similar fluorescence properties to chlorophyllide.

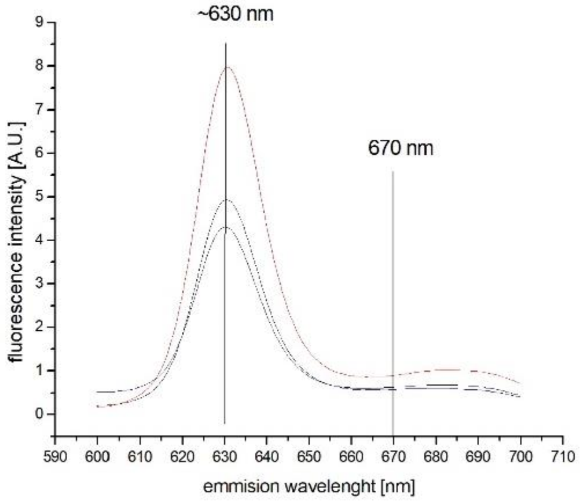

Supplementary Figure S4.3. Fluorescence spectra of acetone extracts measured at room temperature; λ_{exc} = 440 nm.

(3) Analysis of the HPLC chromatograms

The fluorescence detector (λ_{exc}=410 nm, λ_{em}=656 nm) coupled with HPLC was used to exclude the presence of even trace amounts of chlorophylls. Additionally, it confirmed the presence of protochlorophyllide in the samples. Figures below present chromatograms of an etiolated sample, sample undergoing deetiolation, and a sample after 48 hours of deetiolation, respectively.

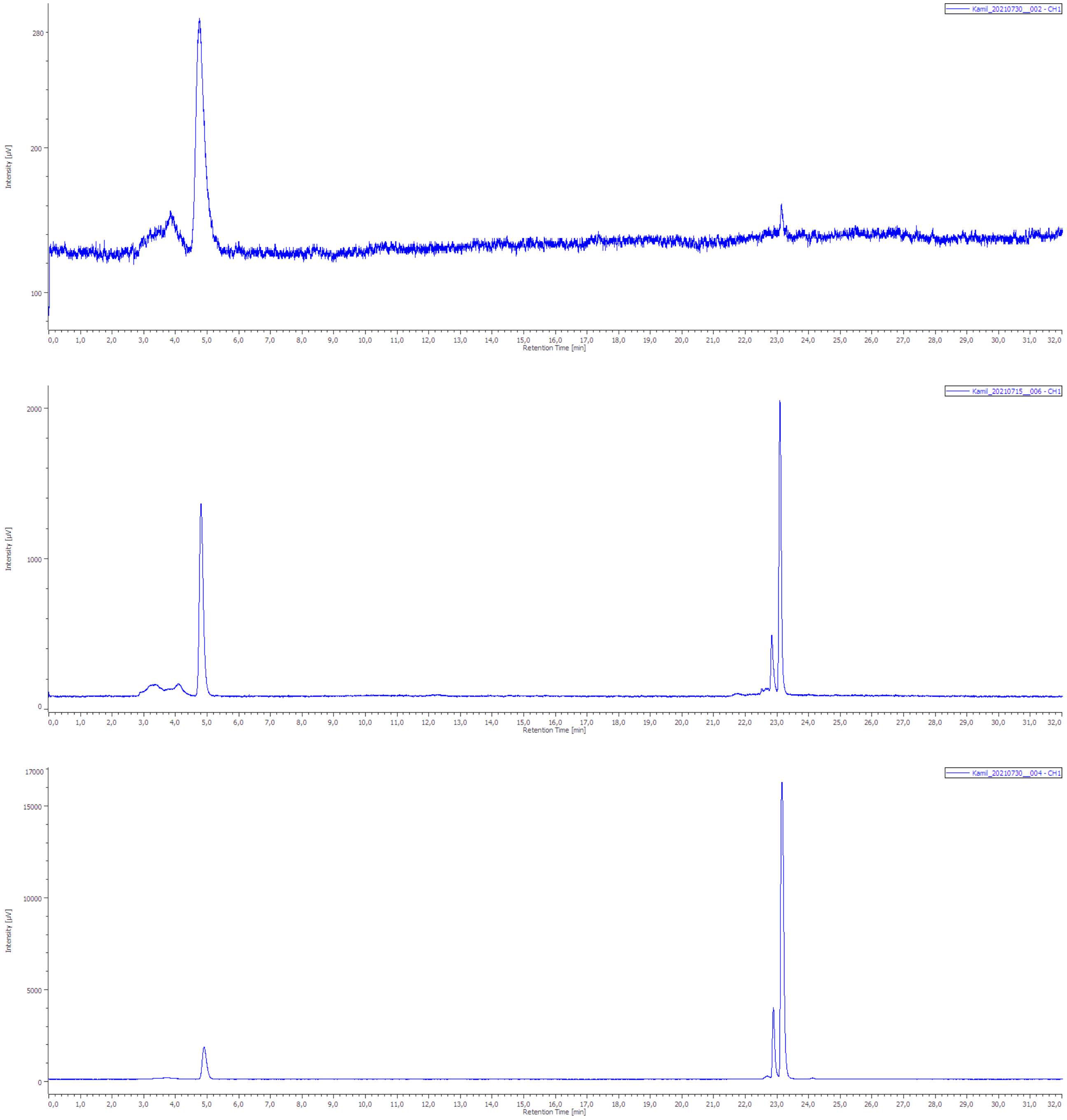

The Pchlid peak is visible around the 5 min. The peaks around the 23 min are Chl *b* and *a*, respectively.

Supplementary data S5. Statistical analysis related to Figure 1

Table S5.1. Statistically significant differences for the length of seedlings shown in the Figure 1B from two-way ANOVA and Tuckey *post-hoc* test.

significance	etiolation time [day]	Type	*versus*	etiolation time [day]	type
P<0.005	3	WT	*vs*	12	WT
P<0.005	3	*lut2* mutant	*vs*	12	WT
P<0.005	4	WT	*vs*	12	WT
P<0.05	6	WT	*vs*	12	WT
P<0.05	4	*lut2* mutant	*vs*	12	*lut2* mutant
P<0.005	6	WT	*vs*	12	*lut2* mutant
P<0.05	4	*lut2* mutant	*vs*	2	WT
P<0.005	8	WT	*vs*	2	WT
P<0.005	6	*lut2* mutant	*vs*	2	*lut2* mutant
P<0.05	8	WT	*vs*	2	*lut2* mutant
P<0.005	6	*lut2* mutant	*vs*	3	WT
P<0.005	8	*lut2* mutant	*vs*	3	WT
P<0.005	6	*lut2* mutant	*vs*	3	*lut2* mutant
P<0.005	8	*lut2* mutant	*vs*	3	*lut2* mutant
P<0.01	6	*lut2* mutant	*vs*	4	WT
P<0.005	8	*lut2* mutant	*vs*	4	WT
P<0.05	8	*lut2* mutant	*vs*	6	WT

Table S5.2. Statistically significant differences for percentage share of the particular categories in the population of seedlings at different age for WT and *lut2* mutant shown in Figures 1D and 1E, from Kruskal-Wallis and *post-hoc* Dunn tests. Ranks A, B, C correspond to seedling categories regarding the curvature of apical hook, as shown in Figure 1C.

**Part 1: Significant differences among ranks for seedlings (WT or lut2 mutant) at certain age.**							
significance	etiolation time [day]	Type	rank	versus	etiolation time [day]	Type	rank
P<0.005	3	WT	A	vs	3	WT	C
P<0.05	4	WT	A	vs	4	WT	C
P<0.01	6	WT	A	vs	6	WT	B
P<0.05	6	WT	A	vs	6	WT	C
P<0.005	8	WT	A	vs	8	WT	B
P<0.005	8	WT	A	vs	8	WT	C
P<0.005	12	WT	A	vs	12	WT	B
P<0.005	12	WT	A	vs	12	WT	C
P<0.005	3	WT	B	vs	3	WT	C
P<0.05	4	WT	B	vs	4	WT	C
P<0.005	8	WT	B	vs	8	WT	C
P<0.005	12	WT	B	vs	12	WT	C
P<0.005	3	*lut2* mutant	A	vs	3	lut2 mutant	C
P<0.01	6	*lut2* mutant	A	vs	6	lut2 mutant	B
P<0.05	8	*lut2* mutant	A	vs	8	lut2 mutant	B
P<0.005	8	*lut2* mutant	A	vs	8	lut2 mutant	C
P<0.005	12	*lut2* mutant	A	vs	12	lut2 mutant	C
P<0.005	3	*lut2* mutant	B	vs	3	lut2 mutant	C
P<0.05	4	*lut2* mutant	B	vs	4	lut2 mutant	C
P<0.005	8	*lut2* mutant	B	vs	8	lut2 mutant	C
P<0.005	12	*lut2* mutant	B	vs	12	lut2 mutant	C
**Part 2: Significant differences for the same ranks and different length of etiolation, for WT and lut2 mutant**							
significance	etiolation time [day]	Type	rank	versus	etiolation time [day]	type	rank
P<0.01	3	WT	A	vs	6	WT	A
P<0.005	3	WT	A	vs	8	WT	A
P<0.005	3	WT	A	vs	12	WT	A
P<0.05	4	WT	A	vs	6	WT	A
P<0.05	4	WT	A	vs	8	WT	A
P<0.01	4	WT	A	vs	12	WT	A
P<0.005	6	WT	A	vs	8	WT	A
P<0.005	6	WT	A	vs	12	WT	A
P<0.005	3	WT	B	vs	8	WT	B
P<0.005	3	WT	B	vs	12	WT	B
P<0.05	4	WT	B	vs	8	WT	B
P<0.05	4	WT	B	vs	12	WT	B
P<0.01	6	WT	B	vs	8	WT	B

$P<0.01$	6	WT	B	vs	12	WT	B
$P<0.01$	3	WT	C	vs	6	WT	C
$P<0.005$	3	WT	C	vs	8	WT	C
$P<0.005$	3	WT	C	vs	12	WT	C
$P<0.05$	4	WT	C	vs	6	WT	C
$P<0.05$	4	WT	C	vs	8	WT	C
$P<0.05$	4	WT	C	vs	12	WT	C
$P<0.01$	6	WT	C	vs	8	WT	C
$P<0.01$	6	WT	C	vs	12	WT	C
$P<0.01$	3	*lut2* mutant	A	vs	6	lut2 mutant	A
$P<0.005$	3	*lut2* mutant	A	vs	8	lut2 mutant	A
$P<0.005$	3	*lut2* mutant	A	vs	12	lut2 mutant	A
$P<0.05$	4	*lut2* mutant	A	vs	8	lut2 mutant	A
$P<0.05$	4	*lut2* mutant	A	vs	12	lut2 mutant	A
$P<0.05$	6	*lut2* mutant	A	vs	8	lut2 mutant	A
$P<0.05$	6	*lut2* mutant	A	vs	12	lut2 mutant	A
$P<0.005$	3	*lut2* mutant	B	vs	8	lut2 mutant	B
$P<0.005$	3	*lut2* mutant	B	vs	12	lut2 mutant	B
$P<0.05$	4	*lut2* mutant	B	vs	8	lut2 mutant	B
$P<0.05$	4	*lut2* mutant	B	vs	12	lut2 mutant	B
$P<0.01$	6	*lut2* mutant	B	vs	8	lut2 mutant	B
$P<0.01$	6	*lut2* mutant	B	vs	12	lut2 mutant	B
$P<0.005$	3	*lut2* mutant	C	vs	8	lut2 mutant	C
$P<0.005$	3	*lut2* mutant	C	vs	12	lut2 mutant	C
$P<0.05$	4	*lut2* mutant	C	vs	8	lut2 mutant	C
$P<0.05$	4	*lut2* mutant	C	vs	12	lut2 mutant	C
$P<0.01$	6	*lut2* mutant	C	vs	8	lut2 mutant	C
$P<0.01$	6	*lut2* mutant	C	vs	12	lut2 mutant	C
**Part 3: Other significant differences, not indicated in part 1 and part 2 above**							
significance	etiolation time [day]	Type	rank	versus	etiolation time [day]	type	rank
$P<0.005$	3	WT	A	vs	8	WT	B
$P<0.005$	3	WT	A	vs	12	WT	B
$P<0.05$	3	WT	A	vs	4	WT	C
$P<0.005$	3	WT	A	vs	8	WT	C
$P<0.005$	3	WT	A	vs	12	WT	C
$P<0.05$	4	WT	A	vs	8	WT	B
$P<0.05$	4	WT	A	vs	12	WT	B
$P<0.05$	4	WT	A	vs	3	WT	C
$P<0.05$	4	WT	A	vs	8	WT	C
$P<0.05$	4	WT	A	vs	12	WT	C
$P<0.01$	6	WT	A	vs	3	WT	B
$P<0.05$	6	WT	A	vs	4	WT	B
$P<0.05$	6	WT	A	vs	3	WT	C
$P<0.01$	6	WT	A	vs	8	WT	C
$P<0.01$	6	WT	A	vs	12	WT	C

P<0.005	8	WT	A	vs	3	WT	B
P<0.05	8	WT	A	vs	4	WT	B
P<0.005	8	WT	A	vs	6	WT	B
P<0.005	8	WT	A	vs	12	WT	B
P<0.05	8	WT	A	vs	4	WT	C
P<0.005	8	WT	A	vs	6	WT	C
P<0.005	8	WT	A	vs	12	WT	C
P<0.005	12	WT	A	vs	3	WT	B
P<0.01	12	WT	A	vs	4	WT	B
P<0.005	12	WT	A	vs	6	WT	B
P<0.005	12	WT	A	vs	8	WT	B
P<0.05	12	WT	A	vs	3	WT	C
P<0.01	12	WT	A	vs	4	WT	C
P<0.005	12	WT	A	vs	6	WT	C
P<0.005	12	WT	A	vs	8	WT	C
P<0.05	3	WT	B	vs	4	WT	C
P<0.005	3	WT	B	vs	8	WT	C
P<0.005	3	WT	B	vs	12	WT	C
P<0.05	4	WT	B	vs	3	WT	C
P<0.05	4	WT	B	vs	8	WT	C
P<0.05	4	WT	B	vs	12	WT	C
P<0.01	6	WT	B	vs	3	WT	C
P<0.05	6	WT	B	vs	4	WT	C
P<0.01	6	WT	B	vs	8	WT	C
P<0.01	6	WT	B	vs	12	WT	C
P<0.01	8	WT	B	vs	6	WT	C
P<0.005	8	WT	B	vs	12	WT	C
P<0.01	12	WT	B	vs	6	WT	C
P<0.005	12	WT	B	vs	8	WT	C
P<0.005	3	*lut2* mutant	A	vs	8	*lut2* mutant	B
P<0.005	3	*lut2* mutant	A	vs	12	*lut2* mutant	B
P<0.05	3	*lut2* mutant	A	vs	4	*lut2* mutant	C
P<0.005	3	*lut2* mutant	A	vs	8	*lut2* mutant	C
P<0.005	3	*lut2* mutant	A	vs	12	*lut2* mutant	C
P<0.05	4	*lut2* mutant	A	vs	12	*lut2* mutant	B
P<0.05	4	*lut2* mutant	A	vs	3	*lut2* mutant	C
P<0.05	4	*lut2* mutant	A	vs	8	*lut2* mutant	C
P<0.05	4	*lut2* mutant	A	vs	12	*lut2* mutant	C
P<0.01	6	*lut2* mutant	A	vs	3	*lut2* mutant	B
P<0.05	6	*lut2* mutant	A	vs	4	*lut2* mutant	B
P<0.05	6	*lut2* mutant	A	vs	3	*lut2* mutant	C
P<0.01	6	*lut2* mutant	A	vs	8	*lut2* mutant	C
P<0.01	6	*lut2* mutant	A	vs	12	*lut2* mutant	C
P<0.005	8	*lut2* mutant	A	vs	3	*lut2* mutant	B
P<0.05	8	*lut2* mutant	A	vs	4	*lut2* mutant	B

P<0.01	8	*lut2* mutant	A	vs	6	*lut2* mutant	B
P<0.05	8	*lut2* mutant	A	vs	6	*lut2* mutant	C
P<0.005	8	*lut2* mutant	A	vs	12	*lut2* mutant	C
P<0.005	12	*lut2* mutant	A	vs	3	*lut2* mutant	B
P<0.05	12	*lut2* mutant	A	vs	4	*lut2* mutant	B
P<0.005	12	*lut2* mutant	A	vs	6	*lut2* mutant	B
P<0.05	12	*lut2* mutant	A	vs	8	*lut2* mutant	B
P<0.05	12	*lut2* mutant	A	vs	6	*lut2* mutant	C
P<0.005	12	*lut2* mutant	A	vs	8	*lut2* mutant	C
P<0.05	3	*lut2* mutant	B	vs	4	*lut2* mutant	C
P<0.005	3	*lut2* mutant	B	vs	8	*lut2* mutant	C
P<0.005	3	*lut2* mutant	B	vs	12	*lut2* mutant	C
P<0.05	4	*lut2* mutant	B	vs	3	*lut2* mutant	C
P<0.05	4	*lut2* mutant	B	vs	8	*lut2* mutant	C
P<0.05	4	*lut2* mutant	B	vs	12	*lut2* mutant	C
P<0.01	6	*lut2* mutant	B	vs	3	*lut2* mutant	C
P<0.05	6	*lut2* mutant	B	vs	4	*lut2* mutant	C
P<0.05	6	*lut2* mutant	B	vs	8	*lut2* mutant	C
P<0.05	6	*lut2* mutant	B	vs	12	*lut2* mutant	C
P<0.005	8	*lut2* mutant	B	vs	12	*lut2* mutant	C
P<0.005	12	*lut2* mutant	B	vs	8	*lut2* mutant	C
P<0.005	3	WT	A	vs	3	*lut2* mutant	C
P<0.05	4	WT	A	vs	4	*lut2* mutant	C
P<0.01	6	WT	A	vs	6	*lut2* mutant	B
P<0.05	8	WT	A	vs	8	*lut2* mutant	B
P<0.005	8	WT	A	vs	8	*lut2* mutant	C
P<0.005	12	WT	A	vs	12	*lut2* mutant	C
P<0.005	3	WT	B	vs	3	*lut2* mutant	C
P<0.05	4	WT	B	vs	4	*lut2* mutant	C
P<0.01	6	WT	B	vs	6	*lut2* mutant	A
P<0.01	8	WT	B	vs	8	*lut2* mutant	A
P<0.005	8	WT	B	vs	8	*lut2* mutant	C
P<0.05	12	WT	B	vs	12	*lut2* mutant	A
P<0.005	12	WT	B	vs	12	*lut2* mutant	C
P<0.005	3	WT	C	vs	3	*lut2* mutant	A
P<0.005	3	WT	C	vs	3	*lut2* mutant	B
P<0.05	4	WT	C	vs	4	*lut2* mutant	A
P<0.05	4	WT	C	vs	4	*lut2* mutant	B
P<0.01	6	WT	C	vs	6	*lut2* mutant	A
P<0.005	8	WT	C	vs	8	*lut2* mutant	A
P<0.005	8	WT	C	vs	8	*lut2* mutant	B
P<0.005	12	WT	C	vs	12	*lut2* mutant	A
P<0.005	12	WT	C	vs	12	*lut2* mutant	B
P<0.005	3	WT	A	vs	8	*lut2* mutant	B
P<0.005	3	WT	A	vs	12	*lut2* mutant	B
P<0.05	3	WT	A	vs	4	*lut2* mutant	C

P<0.005	3	WT	A	vs	8	*lut2* mutant	C
P<0.005	3	WT	A	vs	12	*lut2* mutant	C
P<0.05	4	WT	A	vs	8	*lut2* mutant	B
P<0.05	4	WT	A	vs	12	*lut2* mutant	B
P<0.05	4	WT	A	vs	3	*lut2* mutant	C
P<0.05	4	WT	A	vs	8	*lut2* mutant	C
P<0.05	4	WT	A	vs	12	*lut2* mutant	C
P<0.01	6	WT	A	vs	3	*lut2* mutant	B
P<0.05	6	WT	A	vs	4	*lut2* mutant	B
P<0.05	6	WT	A	vs	12	*lut2* mutant	B
P<0.05	6	WT	A	vs	3	*lut2* mutant	C
P<0.01	6	WT	A	vs	8	*lut2* mutant	C
P<0.01	6	WT	A	vs	12	*lut2* mutant	C
P<0.005	8	WT	A	vs	3	*lut2* mutant	B
P<0.05	8	WT	A	vs	4	*lut2* mutant	B
P<0.005	8	WT	A	vs	6	*lut2* mutant	B
P<0.05	8	WT	A	vs	3	*lut2* mutant	C
P<0.05	8	WT	A	vs	6	*lut2* mutant	C
P<0.005	8	WT	A	vs	12	*lut2* mutant	C
P<0.005	12	WT	A	vs	3	*lut2* mutant	B
P<0.01	12	WT	A	vs	4	*lut2* mutant	B
P<0.005	12	WT	A	vs	6	*lut2* mutant	B
P<0.01	12	WT	A	vs	8	*lut2* mutant	B
P<0.05	12	WT	A	vs	3	*lut2* mutant	C
P<0.05	12	WT	A	vs	6	*lut2* mutant	C
P<0.005	12	WT	A	vs	8	*lut2* mutant	C
P<0.01	3	WT	B	vs	6	*lut2* mutant	A
P<0.005	3	WT	B	vs	8	*lut2* mutant	A
P<0.005	3	WT	B	vs	12	*lut2* mutant	A
P<0.05	3	WT	B	vs	4	*lut2* mutant	C
P<0.005	3	WT	B	vs	8	*lut2* mutant	C
P<0.005	3	WT	B	vs	12	*lut2* mutant	C
P<0.05	4	WT	B	vs	6	*lut2* mutant	A
P<0.05	4	WT	B	vs	8	*lut2* mutant	A
P<0.05	4	WT	B	vs	12	*lut2* mutant	A
P<0.05	4	WT	B	vs	3	*lut2* mutant	C
P<0.05	4	WT	B	vs	8	*lut2* mutant	C
P<0.05	4	WT	B	vs	12	*lut2* mutant	C
P<0.01	6	WT	B	vs	8	*lut2* mutant	A
P<0.005	6	WT	B	vs	12	*lut2* mutant	A
P<0.01	6	WT	B	vs	3	*lut2* mutant	C
P<0.05	6	WT	B	vs	4	*lut2* mutant	C
P<0.01	6	WT	B	vs	8	*lut2* mutant	C
P<0.01	6	WT	B	vs	12	*lut2* mutant	C
P<0.005	8	WT	B	vs	3	*lut2* mutant	A

P<0.01	8	WT	B	vs	12	*lut2* mutant	A
P<0.005	8	WT	B	vs	3	*lut2* mutant	C
P<0.005	8	WT	B	vs	12	*lut2* mutant	C
P<0.005	12	WT	B	vs	3	*lut2* mutant	A
P<0.05	12	WT	B	vs	4	*lut2* mutant	A
P<0.05	12	WT	B	vs	8	*lut2* mutant	A
P<0.005	12	WT	B	vs	8	*lut2* mutant	C
P<0.05	3	WT	C	vs	4	*lut2* mutant	A
P<0.05	3	WT	C	vs	4	*lut2* mutant	B
P<0.01	3	WT	C	vs	6	*lut2* mutant	B
P<0.05	4	WT	C	vs	3	*lut2* mutant	A
P<0.05	4	WT	C	vs	8	*lut2* mutant	A
P<0.05	4	WT	C	vs	12	*lut2* mutant	A
P<0.05	4	WT	C	vs	3	*lut2* mutant	B
P<0.05	4	WT	C	vs	6	*lut2* mutant	B
P<0.01	6	WT	C	vs	8	*lut2* mutant	A
P<0.005	6	WT	C	vs	12	*lut2* mutant	A
P<0.05	6	WT	C	vs	8	*lut2* mutant	B
P<0.01	6	WT	C	vs	12	*lut2* mutant	B
P<0.005	8	WT	C	vs	3	*lut2* mutant	A
P<0.05	8	WT	C	vs	4	*lut2* mutant	A
P<0.01	8	WT	C	vs	6	*lut2* mutant	A
P<0.005	8	WT	C	vs	12	*lut2* mutant	A
P<0.005	8	WT	C	vs	3	*lut2* mutant	B
P<0.05	8	WT	C	vs	4	*lut2* mutant	B
P<0.005	8	WT	C	vs	12	*lut2* mutant	B
P<0.005	12	WT	C	vs	3	*lut2* mutant	A
P<0.05	12	WT	C	vs	4	*lut2* mutant	A
P<0.01	12	WT	C	vs	6	*lut2* mutant	A
P<0.005	12	WT	C	vs	8	*lut2* mutant	A
P<0.005	12	WT	C	vs	3	*lut2* mutant	B
P<0.05	12	WT	C	vs	4	*lut2* mutant	B
P<0.05	12	WT	C	vs	6	*lut2* mutant	B
P<0.005	12	WT	C	vs	8	*lut2* mutant	B
P<0.01	3	WT	A	vs	6	*lut2* mutant	A
P<0.005	3	WT	A	vs	8	*lut2* mutant	A
P<0.005	3	WT	A	vs	12	*lut2* mutant	A
P<0.05	4	WT	A	vs	6	*lut2* mutant	A
P<0.05	4	WT	A	vs	8	*lut2* mutant	A
P<0.05	4	WT	A	vs	12	*lut2* mutant	A
P<0.01	6	WT	A	vs	3	*lut2* mutant	A
P<0.01	6	WT	A	vs	8	*lut2* mutant	A
P<0.01	6	WT	A	vs	12	*lut2* mutant	A
P<0.005	8	WT	A	vs	3	*lut2* mutant	A
P<0.05	8	WT	A	vs	4	*lut2* mutant	A
P<0.01	8	WT	A	vs	6	*lut2* mutant	A

P<0.005	12	WT	A	vs	3	*lut2* mutant	A
P<0.01	12	WT	A	vs	4	*lut2* mutant	A
P<0.005	12	WT	A	vs	6	*lut2* mutant	A
P<0.005	3	WT	B	vs	8	*lut2* mutant	B
P<0.005	3	WT	B	vs	12	*lut2* mutant	B
P<0.05	4	WT	B	vs	8	*lut2* mutant	B
P<0.05	4	WT	B	vs	12	*lut2* mutant	B
P<0.01	6	WT	B	vs	8	*lut2* mutant	B
P<0.01	6	WT	B	vs	12	*lut2* mutant	B
P<0.005	8	WT	B	vs	3	*lut2* mutant	B
P<0.05	8	WT	B	vs	4	*lut2* mutant	B
P<0.01	8	WT	B	vs	6	*lut2* mutant	B
P<0.05	8	WT	B	vs	12	*lut2* mutant	B
P<0.005	12	WT	B	vs	3	*lut2* mutant	B
P<0.05	12	WT	B	vs	4	*lut2* mutant	B
P<0.01	12	WT	B	vs	6	*lut2* mutant	B
P<0.005	3	WT	C	vs	8	*lut2* mutant	C
P<0.005	3	WT	C	vs	12	*lut2* mutant	C
P<0.05	4	WT	C	vs	3	*lut2* mutant	C
P<0.05	4	WT	C	vs	8	*lut2* mutant	C
P<0.05	4	WT	C	vs	12	*lut2* mutant	C
P<0.01	6	WT	C	vs	3	*lut2* mutant	C
P<0.05	6	WT	C	vs	4	*lut2* mutant	C
P<0.01	6	WT	C	vs	8	*lut2* mutant	C
P<0.01	6	WT	C	vs	12	*lut2* mutant	C
P<0.005	8	WT	C	vs	3	*lut2* mutant	C
P<0.05	8	WT	C	vs	4	*lut2* mutant	C
P<0.01	8	WT	C	vs	6	*lut2* mutant	C
P<0.05	8	WT	C	vs	12	*lut2* mutant	C
P<0.005	12	WT	C	vs	3	*lut2* mutant	C
P<0.05	12	WT	C	vs	4	*lut2* mutant	C
P<0.01	12	WT	C	vs	6	*lut2* mutant	C
